# QSAR-ME Profiler 2025: A New Software for QSA(P)R Predictions Supported by Structural Analysis

**DOI:** 10.1021/acs.chemrestox.5c00552

**Published:** 2026-04-22

**Authors:** Nicola Chirico, Arianna Sgariboldi, Marco Evangelista, Ester Papa

**Affiliations:** † QSAR Research Unit in Environmental Chemistry and Ecotoxicology, Department of Theoretical and Applied Sciences, 19045 University of Insubria, via J.H. Dunant 3, 21100 Varese, Italy; ‡ Department of Science and High Technology, University of Insubria, via Valleggio 11, 22100 Como, Italy

## Abstract

Quantitative structure–activity (property) relationships (QSA­(P)­Rs) are an alternative to *in vivo* /*in vitro* experiments. Over the past 20 years, QSA­(P)­Rs targeting various properties and activities have been developed by the QSAR research unit at the University of Insubria. The new software QSAR-ME Profiler 2025 streamlines the application of these and other QSA­(P)­Rs and simplifies the evaluation of the predictions. It provides transparent outputs in tabular and graphical form and innovative solutions, compared to similar software, to quantify prediction uncertainty and applicability domain, while supporting the analysis of structural similarity, the application of customized models, and the compilation of QSAR prediction reporting format.

## Graphical abstract

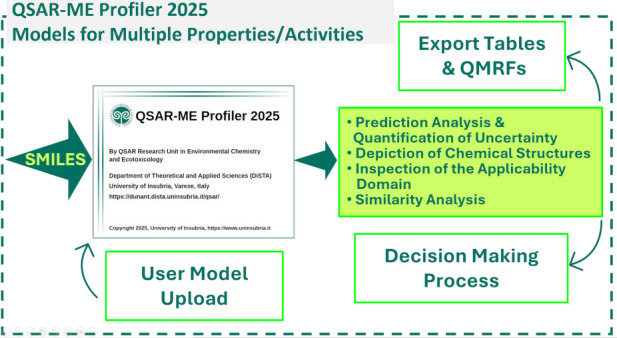

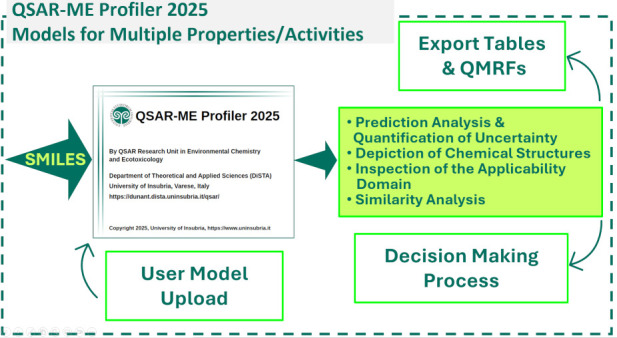

Quantitative structure–activity (property) relationships (QSA­(P)­Rs) are among the new approach methodologies (NAMs) that help assess the toxicological and ecotoxicological hazards of chemicals without the use of animal testing. Models based on QSA­(P)­R are nowadays implemented as *in silico* methodologies and are appealing because they can be applied using common laptop/desktop computers.

A QSA­(P)­R can be described by the expression

endpoint=F(structure)

where *F* is the QSA­(P)­R formula or methodology, “structure” is the input data representing certain aspects of the chemical structure, and “endpoint” is the activity or the property to be modeled. Therefore, the application of a QSA­(P)­R is a relatively straightforward process. However, it should be noted that QSA­(P)­Rs may be developed using limited sets of chemicals, which restricts the range of the user-entered chemical structures (target chemicals) for which reliable predictions can be generated. The limit of reliable application of a QSA­(P)­R model is also known as the applicability domain (AD).^1^ Once the compliance of a target chemical with the AD of the applied QSA­(P)­R has been ascertained, in terms of both chemical structure and predicted value, the reliability of the prediction is further supported by the estimated uncertainty and by a comparison with the experimental measure of the endpoint available for similar compounds within the training set.

If more than one QSA­(P)­R is available for a certain endpoint, the predicted values can be averaged, which is expected to improve reliability and increase the quality of the predictions.^2^ Averaging could also be applied to uncertainties.^3^

In recent years, many tools have become available online to assist QSA­(P)­R-based chemical profiling according to multiple endpoints and are suggested for regulatory purposes.^4^ Among these, EPI Suite,^5^ T.E.S.T (US EPA),^6^ VEGA (IRFMN),^7^ Danish QSAR Database (DTU),^8^ and OECD QSAR Toolbox (OECD - ECHA)^9^ are freely available as online or as standalone tools. However, not all of them provide specific information on prediction reliability and uncertainty or are suitable for tracking models AD and prediction quality easily. Therefore, profiling chemicals in batch, simultaneously for multiple endpoints, analyzing AD and prediction quality, detecting the most similar compounds, and combining predictions (where applicable) may become a task that is overwhelming and prone to error, especially if it should be performed for several QSA­(P)­Rs and/or target chemicals.

To address this scenario, the QSAR Research Unit in Environmental Chemistry and Ecotoxicology at the University of Insubria (Italy) has developed, using the Java language version 21, a new software called QSAR Multiple Endpoint Profiler 2025 (QSAR-ME Profiler 2025), which can be run on different operative systems, for example, Windows, Linux, and MacOS. The software, which is shipped with 98 QSA­(P)­Rs developed by the same research unit over the last 20 years, has been released under proprietary license and is freely downloadable at https://dunant.dista.uninsubria.it/qsar (software section). The source code of the descriptors library, which is part of QSAR-ME Profiler 2025, has been released under an LGPL 2.1 license. These models cover physical–chemical and environmental properties, ecotoxicity, toxicity, and ADME properties. Each QSA­(P)­R includes the corresponding QSAR model reporting format (QMRF), which is a document detailing its compliance with the regulatory requirements.^10^ In addition, predictions, statistics, and results from the evaluation of the AD and similarity analysis can be exported as spreadsheets (Microsoft Excel format.xls) suitable for compiling QSAR prediction reporting format (QPRF) (documents as defined in Annex 2 of the OECD guidance^10^).

A key innovation, compared to other similar software, is that user-defined QSA­(P)­Rs can be added to the QSAR-ME Profiler 2025 and applied for predictions. User-defined QSA­(P)­Rs must be written using XML (Extensible Markup Language), which is text based. By dropping the XML files in a suitable folder and doing some editing to a configuration file, the new models are automatically loaded in QSAR-ME Profiler 2025. Furthermore, potential metabolites, generated by cytochrome P-450 mediated reactions (mammal metabolism), can be detected by Toxtree^11^ and automatically added to the workflow of QSAR-ME Profiler 2025. Figure 1 shows an example of the QSAR-ME Profiler 2025 main screen. All of the available QSA­(P)­Rs are automatically applied to the target chemicals. Structures for the training set chemicals included in each QSA­(P)­Rs and target chemicals are automatically depicted. The results are organized in tables, and the quantification of the AD and of the uncertainty in prediction is automatically performed for regression and classification models. Furthermore, combined predictions can be calculated by clicking on the QSA­(P)­Rs of interest. In addition, the QSA­(P)­Rs, including predictions and data of the target and of the training chemicals, can be browsed in full detail both in tabular and in graphical forms, thus providing further details of the AD compliance of the target chemicals and allowing for the detection of the most similar compounds.

**Figure 1:**
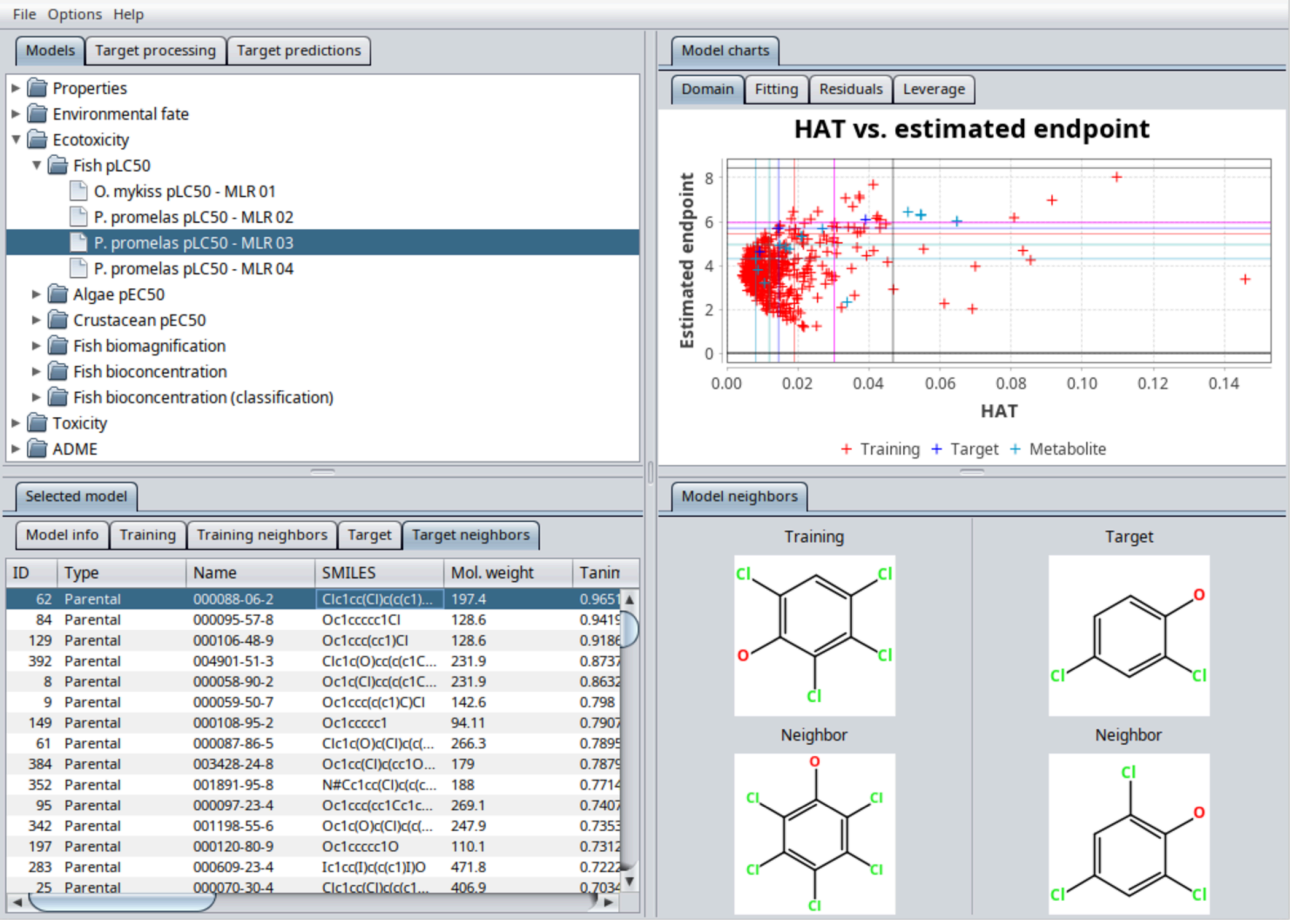
Example of QSAR-ME Profiler 2025 main screen: list of available models, chart for the AD analysis, and neighbors analysis.

## Features

### Available QSA­(P)­Rs

The 98 QSA­(P)­Rs shipped with QSAR-ME Profiler 2025 are organized in groups and subgroups as shown in Table 1. Groups are general categories like “Properties”, “Environmental fate”, or “Ecotoxicity”, while subgroups are QSA­(P)­Rs sharing coherent endpoints like the subgroup “Fish pLC50” or “Algae pEC50” within the “Ecotoxicity” category.

**Table 1:** QSARs Shipped with QSAR-ME Profiler 2025

Group	Subgroup	Available models
Properties	Partition coefficients	1 × Soil organic carbon–water partion^13,14^
Environmental fate	Half-life	1 × Global Half-Life Index (GHLI)^13,15^
	PBT	1 × Insubria PBT index^16^
Ecotoxicity	Fish pLC50	3 × P. promelas,^13,17,18^ 1 × O. mykiss^18^
	Algae pEC50	2 × P. subcapitata^17,18^
	Crustacean pEC50	1 × D. magna^17^
	Fish biomagnification	1 × Dietary biomagnification factor (BMF)^19^
	Fish bioconcentration	1 × Dietary bioconcentration factor (BCF)^20^
	Fish bioconcentration (classification)	1 × Dietary bioconcentration factor (BCF)^20^
Toxicity	Human transthyretin disruption	1 × ANSA T4-hTTR competing potency^21^ 1 × FITC-T4 T4-hTTR competing potency^21^ 1 × RLBA T4-hTTR competing potency^21^ 1 × PFAS T4-hTTR competing potency^21^
	Human transthyretin disruption (classification)	1 × PFAS hTTR disruption^22^
ADME	Fish biotransformation half-life	2 × Metabolic biotransformation^2^
	Human biotransformation half-life	4 × Whole-body^23^
	Human total elimination half-life	1 × Whole body^23^
	Human microsomes clearance	32 × *In vitro* clearance^24^
	Human hepatocytes clearance	8 × *In vitro* clearance^24^
	Rat microsomes clearance	13 × *In vitro* clearance^24^
	Rat hepatocytes clearance	6 × *In vitro* clearance^24^
	Mouse microsomes clearance	14 × *In vitro* clearance^24^

QSAR-ME Profiler 2025 supports multiple linear regression (MLR) and linear discriminant analysis (LDA).

### Prediction Averages and Uncertainties

The prediction interval (i.e., the uncertainty of individual chemicals MLR predictions), is calculated as^12^ σ = $\pm t_{\mathrm{stud}\;\alpha /2}\cdot s\cdot \sqrt{1+h_{ii}}$ where *t*_stud α/2_ is t-student calculated for α/2 = 0.025, *s* is $\sqrt{\frac{\underset{n}{\sum }{\left(y_{n}-{ŷ}_{n}\right)}^{2}}{n-p-1}}$ where *n* is the number of the training set chemicals, and *p* the number of descriptors, and *h*_*ii*_ is the leverage value (see Structural Applicability Domain, where for target chemicals the corresponding *x*_*i*_ has to be used). As a side note, prediction intervals are expected to be relatively wide because the single item random variation is added to the corresponding confidence interval.

As reported by Taylor,^3^ combined measures can be calculated as weighted averages. For each prediction, the weight is calculated as *w* = 1/σ^2^, where σ^2^ is the square of the corresponding uncertainty. For *N* predictions, weighted average is calculated as $\frac{\sum w_{i}{ŷ}_{i}}{\sum w_{i}}$ and the corresponding uncertainty as $\frac{1}{\sqrt{\sum w_{i}}}$ , where *i* = 1, ..., *N* and *ŷ*_*i*_ is the *i*th predicted value.

Concerning LDA (linear discriminant analysis) predictions, uncertainty is evaluated by the Shannon entropy, calculated as $-\underset{n}{\sum }p\left(x_{n}\right)\log p\left(x_{n}\right)$ , where *p* is the posterior probability of the event under scrutiny.

An example of the QSAR-ME Profiler 2025 output table, reporting averaged predictions (consensus analysis), is shown in Figure 2.

**Figure 2:**
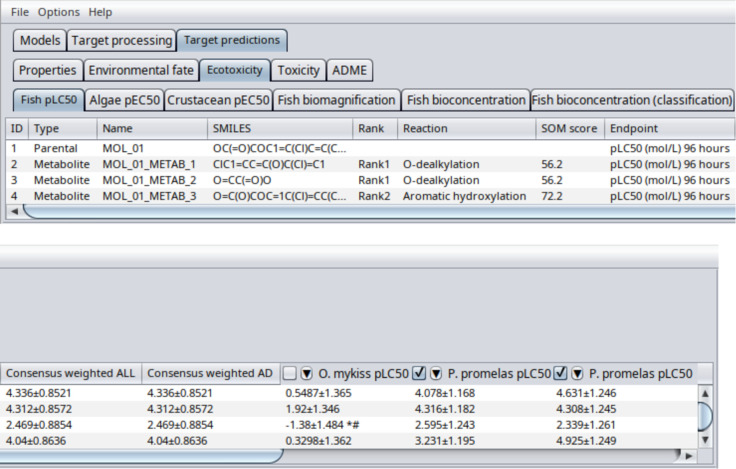
Consensus analysis example from target predictions.

### Structural Applicability Domain

For MLR QSA­(P)­Rs, a chemical is considered out of the AD if its leverage value is greater than or equal to 3·*p*′/*n*, where *p*′ is the number of descriptors +1 and *n* is the number of chemicals in the training set. The leverage value is calculated as *h*_*ii*_ = *x*_*i*_·(*X*·*X* ^*T*^)^−1^·*x*_*i*_^*T*^, where *X* is the training descriptors matrix and *i* the chemical under scrutiny. For LDA QSA­(P)­Rs, a chemical is considered out of the AD if the cosine similarity coefficient^25^ is below the 95th percentile of all training scores.^26^

### End Point Applicability Domain

An MLR prediction is considered out of the AD^1^ of the end point if the standardized residual $r_{i}^{\prime }=\frac{r_{i}}{s\cdot \sqrt{1-h_{ii}}}$ is greater than 2.5 standard deviation units and if the prediction falls outside the experimental range of the training set. Furthermore, for each QSA­(P)­R, the training set minimum and maximum prediction intervals (see Prediction Averages and Uncertainties) define the uncertainty. If the prediction interval of a target chemical is outside this range, then the chemical is arbitrarily considered out of the AD.

Classification models AD can be defined by class probabilities.^25^ An LDA prediction is considered out of the AD if the posterior probability of being (or being not) the event is between 0.25 and 0.75.

### Neighbor Detection

Structural similarity among chemicals, used to identify neighbors, is measured as a distance index calculated using the molecular fingerprint^27^ of two chemicals. The smaller the distance between the two chemicals, the more similar they are considered. Available indexes are Tanimoto, dice, and cosine,^27^ while available fingerprints are Pubchem,^28^ E-State,^29^ Klekota and Roth,^30^ Klekota and Roth count, substructure^31^ and substructure count. As different pairs of index-fingerprint typically lead to different neighbors, the best pair to use is left to the expert’s judgment.

### Descriptor Calculation

As was mentioned before, the QSA­(P)­Rs included in QSAR-ME Profiler 2025 were developed using descriptors calculated by PaDEL-Descriptor version 2.21.^32^ QSAR-ME Profiler 2025 descriptors library, which is based on CDK version 2.11,^33^ calculates 40 descriptor categories (which include 1D and 2D descriptors), 5 fingerprints categories and aims for best reproducibility of the descriptors calculated using PaDEL-Descriptor (which is based on CDK version 1.4). In particular, the QSAR-ME Profiler 2025 library adapts, from the PaDEL-Descriptor source code, the atomic constants table, molecular nitro and aromatic standardization, SMARTS table for MLFER descriptors, and standardization of nitro group for extended topochemical index descriptors. In addition, fragments and related logic for calculation of H-Bond Molconn electrotopological state index descriptors are also deduced from the PaDEL-Descriptor source code. Some peculiarities of PaDEL-Descriptors calculations are also included to improve reproducibility. Details can be found in the QSAR-ME Profiler 2025 descriptor calculation library source code, which is published on our Web site (https://dunant.dista.uninsubria.it/qsar) under an LGPL 2.1 license.

### Metabolite Detection and ADME QSARs

Metabolites from cytochrome P-450 mediated reaction QSARs can be automatically detected by the Toxtree software,^11^ which is shipped with QSAR-ME Profiler 2025 and is run as an external process. Parental chemicals metabolic reactions can be used to automatically select the pertinent *in vitro* clearance QSARs, which cover rat, mouse, and humans (see Table 1). These QSARs were developed using training sets whose chemicals were selected according to the most probable cytochrome P-450 mediated reactions detected by Toxtree; therefore, they should be applied according to the metabolic reaction expected for the target chemical (either automatically detected by Toxtree or manually selected by the user). Originally, these QSARs were developed in the context of the ECO44 project^34^ leading to a software called IVBP-Suite^24^ devoted to this task. The performance of *in vitro* clearance QSARs was subsequently successfully tested, using IVBP-Suite, in a recent collaboration with the US-EPA.^35^ The human biotransformation half-life QSARs were also successfully tested in the same paper using QSARINS-Chem standalone version.^36^

## Conclusions

QSAR-ME Profiler 2025 is a new, freely available, multiplatform software which automates, streamlines, and simplifies the application of about 100 QSA­(P)­Rs targeting endpoints of toxicological and ecotoxicological interest, which can support environmental risk assessment procedures or the screening of chemicals and chemical alternatives. Functionalities of this software include the automated calculation of predictions for MLR and LDA models and the checking of the compliance of predictions and of the molecular structures with the AD of the QSA­(P)­Rs. In particular, the predictions can be explored in full detail in the context of the corresponding QSA­(P)­Rs, in both graphical and tabular formats. Moreover, predictions are automatically grouped and combined in suitable tables, which can be used to compile QPRF reports. The software includes key innovations compared to most of the similar QSAR platforms , such as the automatic profiling (in batch) of parent compounds and their metabolites for all the properties and activities predicted by the software. Other innovations include the quantification of prediction uncertainty, in both regression and classification, and the analysis of the structural domain extended to the most similar compounds in the QSA­(P)­R training set. Furthermore, user-defined QSA­(P)­Rs can be added to suit specific needs of the users, such as to apply models based on descriptors calculated by a software of choice or on experimental measures. This last feature is helpful for profiling materials (e.g., nanomaterials or micro- and nanoplastics), which are mostly characterized using experimental measurements, as well as for other chemical entities (e.g., mixtures), whose molecular structure cannot be described using traditional molecular descriptors. Users can therefore create their own MLR or LDA models based on *ad hoc* generated theoretical or experimental descriptors. They can add user-defined models to QSAR-ME 2025 Profiler as external QSARs, analyze their AD and statistical quality, and apply them to profile target chemicals. The software is freely available at https://dunant.dista.uninsubria.it/qsar, in the software section. Detailed instructions on how to install and run QSAR-ME Profiler 2025 are provided in the Supporting Information.

## Supplementary Material



## Notes

Detailed instructions on how to install and run QSAR-ME Profiler 2025 (PDF)
