# Supplementary material for: QSAR-ME Profiler 2025: A New Software for QSA(P)R Predictions Supported by Structural Analysis
Source: Chem Res Toxicol. 2026 Apr 22;39(5):813–8. doi: 10.1021/acs.chemrestox.5c00552 (PMC13188051; doi:10.1021/acs.chemrestox.5c00552)
Supplement: Supplementary file 1 [file tx5c00552_si_001.pdf]

Supporting Information for the manuscript

QSAR-ME Profiler 2025: a new software for QSA(P)R predictions supported by structural analysis

Nicola Chirico,[1*] Arianna Sgariboldi[1,2], Marco Evangelista[1,2] and Ester Papa[1*]

[1] *QSAR Research Unit in Environmental Chemistry and Ecotoxicology, Department of Theoretical and Applied Sciences, University of Insubria, via J.H. Dunant 3, 21100, Varese (Italy)*

[2] *Department of Science and High Technology, University of Insubria, via Valleggio 11, 22100 Como (Italy)*

E-mail: nicola.chirico@uninsubria.it; ester.papa@uninsubria.it

*** QSAR-ME Profiler 2025 is actively maintained. Please check our website (**https://dunant.dista.uninsubria.it/qsar/ **) for updates and bug fixes.**

How to cite: Any work utilizing QSARME-Profiler 2025 must cite the present manuscript as the primary reference.

**** QSAR-ME Profiler 2025 requirements:**

QSAR-ME Profiler 2025 requires JavaTM SE 21 runtime environment or a more recent version.

***** QSAR-ME Profiler 2025 installation:**

> Unzip the downloaded file in a folder of choice

To run the software:

> double click on the **QSAR-ME-Profiler.jar** icon

- alternatively -

> Open a terminal pointing to the QSAR-ME Profiler main folder

> Type in: **java -jar QSAR-ME-Profiler.jar**

> Press Enter to run the software
